# Pharmacogenetics of Complement Factor H Y402H Polymorphism and Treatment of Neovascular AMD with Anti-VEGF Agents: A Meta-Analysis

**DOI:** 10.1038/srep14517

**Published:** 2015-09-28

**Authors:** Guohai Chen, Radouil Tzekov, Wensheng Li, Fangzheng Jiang, Sihong Mao, Yuhua Tong

**Affiliations:** 1 Department of Ophthalmology, Quzhou People’s Hospital, Quzhou, Zhejiang, PR, China; 2 The Roskamp Institute, Sarasota, Florida, USA; 3 Department of Ophthalmology, University of South Florida, Tampa, Florida, USA; 4 Xiamen Eye Center of Xiamen University, Xiamen, Fujian, PR, China

## Abstract

The purpose of this study is to investigate whether the Y402H polymorphism (rs1061170, a T-to-C transition at amino acid position 402) in the complement factor H (CFH) gene have a pharmacogenetics effect on the anti-vascular endothelial growth factor (VEGF) treatment for neovascular age-related macular degeneration (AMD). We performed a meta-analysis using databases including PubMed and EMBASE to find relevant studies. 13 published association studies were selected for this meta-analysis, including 2704 patients. For the CFH Y402H polymorphism, anti-VEGF treatment was much less effective in AMD patients with the CFH CC genotype (CC versus TT: odds ratio (OR) = 55, 95% confidence interval (CI), 0.31 to 0.95, P = 0.03; CC versus CT: OR = 0.60, 95% CI, 0.40 to 0.91, P = 0.02; and CC versus CT + TT: OR = 0.59, 95% CI, 0.38 to 0.90, P = 0.02, respectively). In subgroup analysis, CFH Y402H polymorphism was more likely to be a predictor of response for Caucasians (CC versus CT+TT: OR = 0.63, 95% CI, 0.42 to 0.95, P = 0.03). In conclusion, pharmacogenetics of CFH Y402H polymorphism may play a role in response to anti-VEGF treatment for neovascular AMD, especially for Caucasians.

Age-related macular degeneration (AMD) is the leading cause of irreversible blindness in people aged over 50 in the developed world^1^. Although the neovascular form of AMD accounts for only ~20% of all AMD cases, it is responsible for almost 90% of the severe vision loss associated with this disease^2^. It has been demonstrated that vascular endothelial growth factor (VEGF), a signal protein that stimulates vasculogenesis and angiogenesis, plays a key role in formation of neovascularization in AMD^3^,^4^,^5^. Intravitreal injections of anti-VEGF agents, such as the monoclonal antibody fragment ranibizumab (Lucentis, Genentech Inc., San Francisco, CA) and the monoclonal antibody bevacizumab (Avastin, Genentech Inc., San Francisco, CA), are currently considered part of the standard treatment regimen for neovascular AMD^6^. Several years of clinical application of these two drugs have shown a broad range of responses. While most patients experience considerable and sustained improvement in their visual acuity and resolution of the macular edema with long-term treatment, a substantial fraction experience further deterioration of visual acuity and/or persistent macular edema despite intensive and regular treatment^7^. One possible reason for this phenomenon may be a difference in the genetic background between patients who experience improvement and those who do not^8^,^9^.

Genetic factors play an important role in the development of AMD^10^. For example, the single nucleotide polymorphism Y402H (rs1061170, a T-to-C transition at amino acid position 402) in the gene encoding complement factor H (CFH) is recognized as an important one^11^,^12^,^13^. Studies in mostly Caucasian populations showed that possession of at least one histidine at position 402 (CT genotype) increases the risk of AMD ~2.5-fold, while CC genotype increases the risk by ~6-fold and may account for large portion (up to 50%) of the attributable risk of AMD^13^,^14^,^15^. A meta-analysis of genomic association studies in Asian population showed similar, although less pronounced risk (1.97-fold risk of CT genotype and 8.8% attributable risk of AMD)^16^. Additional independent genetic factors, such as mutations in age-related maculopathy susceptibility 2 (ARMS2), C3, C2 and other genes may also play a role^17^.

Although the role of CFH Y420H polymorphism in the overall risk for developing any form of AMD in general and neovascular AMD in particular is well-established, there is still some controversy about its role in the response to anti-VEGF treatment. Thus, patients with the CFH Y420H CC genotype had a lower visual acuity outcome in one study^18^, a better visual acuity outcome in another^19^, while a third study concluded that there was no association between visual acuity outcome and this genotype^20^.

To the best of our knowledge, only one report conducted a meta-analysis focusing on the relationship between the CFH Y402H polymorphism and treatment response of neovascular AMD, indicating that CFH Y402H polymorphism might be associated with treatment response outcome in neovascular AMD^21^. However, this meta-analysis was limited in scope, as it included in the analysis several forms of treatments, including anti-VEGF agents, photodynamic therapy and antioxidants/zinc, and it included only six trials using anti-VEGF treatment as monotherapy (808 patients). Not surprisingly, the authors concluded that the association between Y402H and the positive therapy outcome is not very strong. As more recent relevant data are now available, we decided to conduct an independent assessment of the literature and to undertake a new meta-analysis in order to get a more convincing and precise conclusion about the relationship between the CFH Y402H polymorphism and the response to anti-VEGF treatment for neovascular AMD.

## Results

### Overall characteristics of selected studies and quality assessment

**Figure 1:**
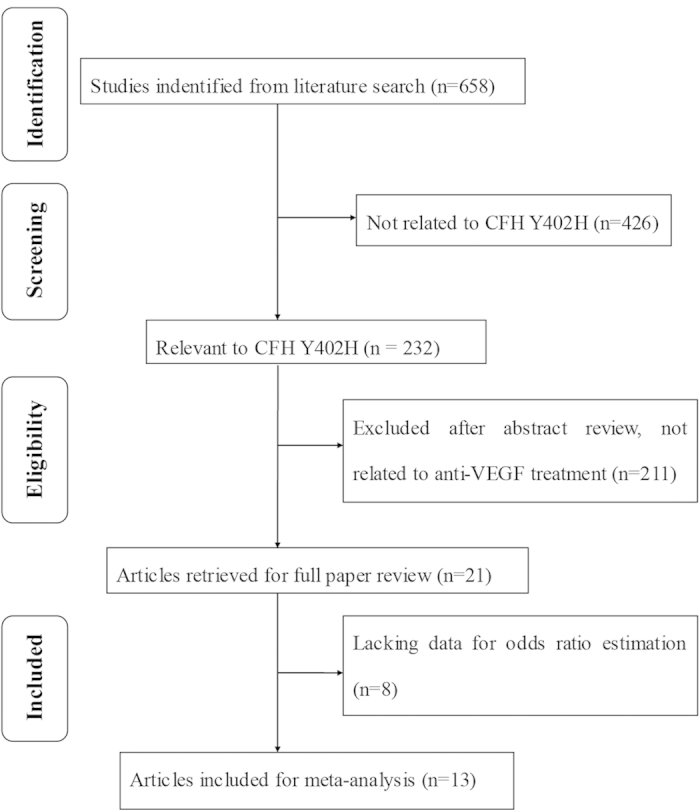
Flow diagram of studies included in this meta-analysis. CFH, complement factor H; VEGF, vascular endothelial growth factor.

A total of 658 articles were initially identified. Of these, 645 were rejected according to the exclusion criteria listed above. Hence, 13 studies were included in this meta-analysis^18^,^19^,^22^,^23^,^24^,^25^,^26^,^27^,^28^,^29^,^30^,^31^,^32^. Figure 1 provides a flow diagram of the search procedure and results. In total, there were 2704 patients included in the meta-analysis. Regarding ethnicity, nine studies included mostly Caucasians, two studies included mostly East Asians, and the ethnical background of the study population in the remaining two studies was unknown. According to the Newcastle-Ottawa Scale (NOS) used for quality assessment, two studies had moderate quality scores of 6, while 11 studies had high quality scores of 7 or 8 (Table 1). The average score of all studies included in the analysis was 7.15. The majority (7 out of 13) studies used ranibizumab only, while 3 studies used either ranibizumab or bevacizumab and 3 studies used bevacizumab only. In total, there were 712 patients with the CFH Y420H CC genotype, 1216 patients with CT genotype, and 776 patients with TT genotype. The frequency of variant C-allele of CFH Y402H in this analysis was 48.8%. Regarding ethnicity, the frequency of variant C-allele in Caucasians was 53.9%. In contrast, a much lower average frequency of the C-allele was reported in the two studies with mostly East Asian populations (11.6%). The genotype distributions of CFH Y402H for all studies are summarized in Table 2.

### Genotype contrast

We calculated a pooled OR based on genotype contrast. The results of meta-analysis for the CFH Y402H polymorphism and treatment response of neovascular AMD with anti-VEGF agents are summarized in Table 3. For the CFH Y402H polymorphism, anti-VEGF treatment was much less effective in AMD patients with the CFH CC genotype (CC versus TT: odds ratio (OR) = 0.55, 95% confidence interval (CI), 0.31 to 0.95, P = 0.03; CC versus CT: OR = 0.60, 95% CI, 0.40 to 0.91, P = 0.02, respectively). However, heterozygous (CT genotype) was not associated with altered treatment response (CT versus TT: OR = 0.93, 95% CI, 0.68 to 1.28, P = 0.65). When we divided the patients according to ethnicity (Caucasians vs. East Asians), CC genotype was also associated with a reduced response to treatment of neovascular AMD in Caucasians (CC versus TT: OR = 0.68, 95% CI, 0.51 to 0.90, P = 0.008; CC versus CT: OR=0.61, 95% CI, 0.38 to 0.96, P = 0.03, respectively), but not in East Asians (CC versus TT: OR = 0.90, 95% CI, 0.18 to 4.55, P = 0.90; CC versus CT: OR = 1.23, 95% CI, 0.24 to 6.16, P = 0.80, respectively).

### Genetic model

**Figure 2:**
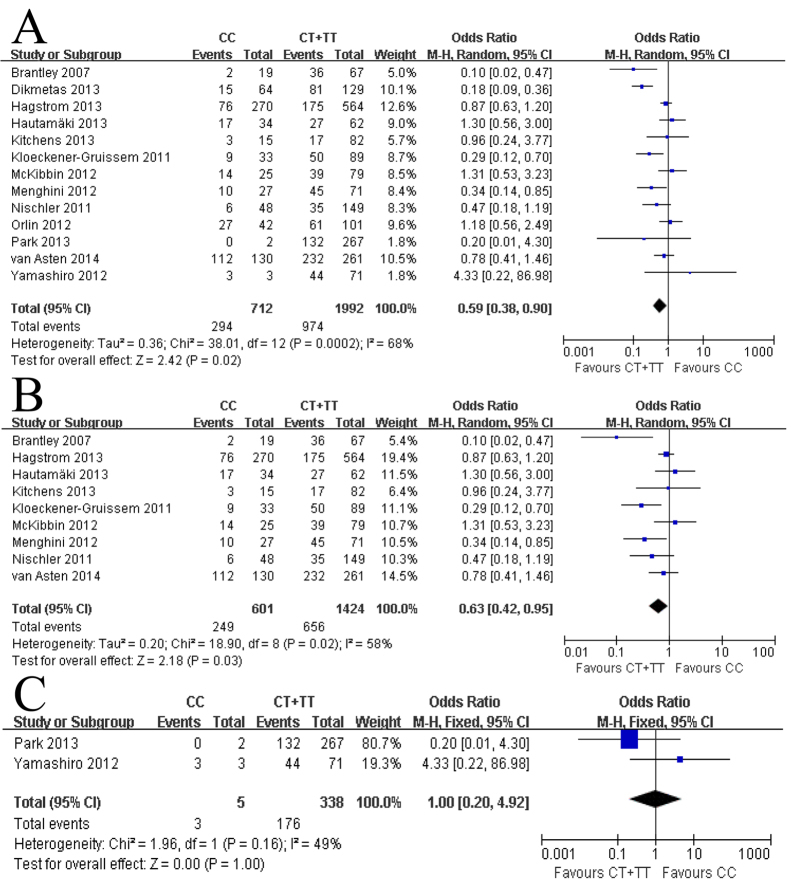
The association between complement factor H Y402H and treatment response of neovascular AMD based on the recessive model. (**A**) Ovearall, (**B**) Caucasians, and (**C**) East Asians. M-H, Mantel-Haenszel statistics; CI, confidence interval.

In this analysis, the presence of CC versus TT genotype and CC versus CT genotype had a significant effect on the improved outcome as a result of anti-VEGF therapy (P = 0.03 and P = 0.02, respectively), while the presence of the CT genotype versus TT did not have a significant effect (p = 0.65). The genetic model indicating mode of inheritance is most likely to be recessive, which compares the CC genotype with the combination of CT and TT genotypes. In the contrasts of the CC versus CT + TT model, the patients with the CC genotype appeared to be associated with a reduced response to anti-VEGF treatment for neovascular AMD (OR = 0.59, 95% CI, 0.38 to 0.90, P = 0.02). In this comparison heterogeneity was also identified and thus, a random-effects model was applied to the data (Fig. 2A). In a subgroup analysis, CFH Y402H polymorphism was more likely to be a predictor of anti-VEGF treatment response for Caucasians (OR = 0.63, 95% CI, 0.42 to 0.95, P = 0.03). Similarly to the main analysis, heterogeneity was identified in this analysis too and a random-effects model was applied to the data (Fig. 2B). In contrast, no heterogeneity was observed for East Asians (OR = 1.00, 95% CI, 0.20 to 4.92, P = 1.00) (Fig. 2C). Harbord’s test and Peter’s test indicated no statistically significant evidence of publication bias for overall studies in the recessive model (P = 0.62 and P = 0.34, respectively).

### Sub-analysis: effect on improvement of visual acuity

**Figure 3:**
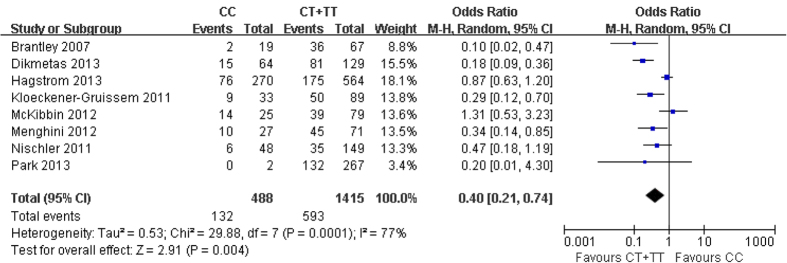
The association between complement factor H Y402H and treatment response of neovascular AMD based on studies that define a positive outcome only as improvement in visual acuity. M-H, Mantel-Haenszel statistics; CI, confidence interval.

One limitation of the analysis when all trials were included is the heterogeneity of the positive treatment outcome, as shown in Table 2. Thus, 10 of the 13 studies included define a positive outcome from anti-VEGF therapy as improvement in visual function (visual acuity), while three studies define it as an improvement in retinal morphology (resolution of macular edema). Furthermore, heterogeneity exists even within the groups of studies based on functional outcome, where most studies define positive outcome as gain in visual acuity, but two studies define it either as visual acuity improved or unchanged^29^ or as decreased loss of visual acuity^31^. To overcome the problem of heterogeneity in outcome definition, we decided to conduct a sub-analysis of studies that define a positive outcome only as improvement in visual acuity. Eight such studies including a total of 1903 patients were selected^18^,^19^,^22^,^23^,^26^,^27^,^28^,^30^. The results of this sub-analysis demonstrated a stronger relationship between the presence of CC polymorphism and a positive visual function outcome after anti-VEGF therapy. Thus, in the comparison CC vs. TT genotype, the OR decreased from 0.55 (when all studies included, Table 3) to 0.36 (CI 0.16–0.82, p = 0.02); similarly, in the comparison CC vs. CT, OR decreased from 0.60 to 0.40 (CI 0.23–0.72, p = 0.002), while the comparison between CT and TT remained practically unchanged at OR = 0.97 (CI 0.61–1.53, p = 0.88). When a comparison was done between the presence of CC genotype versus the presence of either CT or TT genotype (CT+TT), the OR also decreased from 0.59 (all studies, Fig. 2A) to 0.40 (CI 0.21–0.74) and the statistical significance increased substantially, from p = 0.02 to p = 0.004 (Fig. 3).

## Discussion

Pharmacogenetics examines the impact of genetic variation on the response to drugs. It has been suggested that genetic factors may influence response to anti-VEGF treatment in neovascular AMD^8^,^9^. Indeed, as our analysis demonstrates, 41.3% of patients harboring a homozygous risk allele genotype (CC) at the CFH Y402H locus showed good response with anti-VEGF treatment compared with 48.9% of heterozygotes (CT) and wild-type homozygotes (TT), and the result was statistically significant, indicating that anti-VEGF treatment was less effective in AMD patients with the CFH CC genotype. This relationship was strengthen further and became highly significant when a sub-analysis was conducted including studies with a visual acuity positive outcome criterion. Notably, in the sub-analysis, the majority of the included studies (six out of the seven) were of good quality and only one study was of medium quality. Specifically, patients harboring the CC genotype may have about half the chance to improve their visual acuity compared to patients with either the CT or the TT variant after at least 6 months of ranibizumab or bevacizumab therapy. This finding indicates that genetic testing may play a useful role in predicting the outcome of anti-VEGF therapy and, thus have clinical utility, despite the doubts raised after analysis of negative results^9^.

The complement system, which is part of the immune system, plays an important role in inflammation^33^. Some of the major components of the complement cascade like complement factors C3, C5, and C5b-9 complex, have all been detected in drusen, indicating a potential role of the complement system in the pathogenesis of AMD^34^. CFH is capable both of inhibiting the cleavage of C3 to C3a and C3b and of inactivating already existing C3b, and thus it is a critical negative regulator of complement activation^35^. This inhibitory activity is influenced by binding of C-reactive protein (CRP), which enhances the affinity for C3b and leads to the suppression of complement activity^36^. The CFH Y402H polymorphism is located within a binding site for heparin and CRP^37^. Therefore, changes in this region of the molecule may result in a malfunctioning CFH that is not able to inhibit this complement cascade properly^38^. Previous studies have demonstrated that the Y402H polymorphism is associated with reduced affinity of CFH to CRP^39^. Notably, patients with AMD harboring a homozygous risk allele genotype (CC) have increased levels of CRP in the serum and choroid^40^. This aberrant activation of the complement cascade may lead to an enhanced local inflammatory response, which ultimately may lead to increased local levels of VEGF and resulting neovascularization. It was demonstrated in experimental studies that complement factors C3a and C5a induce VEGF expression in retinal pigment epithelial cells^41^. Furthermore, mouse choroidal neovascularization was reduced when inhibiting the alternative pathway of complement activation^42^. Lee *et al.* found that patients with the CC genotype required had a 37% significantly higher risk of requiring additional ranibizumab injections over the first 9 months^20^.

Based on the current analysis, we propose that patients harboring the CFH CC genotype experience a reduced response to anti-VEGF treatment. When a positive response to treatment is defined more narrowly as whether or not an improvement in visual acuity occurs, the difference in response based on CC vs. CT/TT genotype becomes highly significant. Although the exact mechanism underlying an association between CFH genotype and anti-VEGF treatment response is currently unknown, it can be hypothesized that it may involve an enhanced inflammatory response due to an aberrant complement cascade behavior and an inferior capacity of the immune system to downregulate VEGF levels in the retina. As a result, patients with the homozygous CHF Y402H genotype (CC) and, to a much lesser extent, patients with the heterozygous genotype (CT) are likely to have a less favorable response to anti-VEHF treatment, and may require additional applications or switching to different anti-VEGF agents.

Previous meta-analyses showed that the frequency of CFH Y402H C-allele is high in Caucasians (50~58%)^21^, but low in Asian populations (5.1%)^16^. In line with previous reports, this analysis estimates the reported frequency of C-allele in Caucasians at 53.9%, whereas, the frequency in East Asian population was found to be somewhat higher than reported before at 11.6%. As the frequency of the CC genotype for CFH Y402H is low in East Asian patients compared to Caucasian patients, the influence of risk allele homozygosity is hard to evaluate properly in East Asian patients due to the small size of the relevant studies in this meta-analysis and a potential association should be further evaluated in the future when more results from more studies, including a large number of patients from mostly East Asian populations are reported.

This work may have some limitations. First, we cannot fully exclude publication bias. It is possible that some works, especially those published in languages other than English may have been missed. Second, a potential source of heterogeneity is different trial duration and different definition of improved anti-VEGF treatment response, the results should be interpreted with caution. Third, because of the complex nature of AMD, it is unlikely that a single nucleotide polymorphism in a single gene would be the only one associated with an increase in AMD risk and treatment response, without consideration of other polymorphic susceptible genes. For example, the ARMS2 and the VEGF-A genes could be harboring polymorphisms that can have an additional or separate effect on anti-VEGF treatment efficacy. To date, very few studies considered the cumulative effect of risk alleles in multiple genes (CFH, ARMS2, VEGF-A, etc.) with inconsistent results^9^,^43^. Another limitation is that in this meta-analysis, only two ethnic backgrounds were considered (Caucasians and East-Asians). Thus future studies will need to expand this to other backgrounds when data become available.

In conclusion, this is the first detailed meta-analysis to focus on the influence of genetic background on anti-VEGF treatment results. Our analysis provide evidence that pharmacogenetics of CFH Y402H polymorphism likely play a role in the frequency of the positive outcome to anti-VEGF treatment for neovascular AMD, especially in Caucasians and when improvement in visual acuity is defined as a primary outcome measure. Additional prospective studies with larger sample sizes would be helpful to confirm this association and study the influence of other genotypic variations to the treatment of choice.

## Methods

### Search strategy

We conducted searches of PubMed and Embase, using the terms *(“complement factor H” or “CFH”’) and (“age-related macular degeneration” or “AMD”)*. A manual search was performed by checking the reference lists of original reports and review articles to identify studies not yet included in the computerized databases. The final search was carried out on January 25, 2015, without restrictions regarding publication year or language.

### Inclusion and exclusion criteria

Articles were considered eligible for inclusion in the meta-analysis if the studies met the following inclusion criteria: (1) evaluating the relationship between the CFH Y402H polymorphism and the response to anti-VEGF treatment for neovascular AMD, (2) Independent retrospective or prospective association study, and (3) With sufficient available data to estimate an OR with 95% CI. Abstracts from conferences, full texts without raw data available for retrieval, duplicate publications, letters, and review articles were excluded.

### Data extraction

The data were extracted independently by two reviewers (G.H.C. and W.S.L.). Disagreement was resolved by discussion. The information extracted from each study included the authors of each study, the year of reported, information on study design, location and ethnicity of the trial, treatment modality, number of subjects, duration of the study, and genotype distributions.

### Quality assessment

We assessed quality of included studies by a modified checklist based on the NOS^44^, in which a study was judged on three categories: selection (four items, one star each), comparability (one item, up to two stars), and exposure/outcome (three items, one star each). A nine-point scale of the NOS (range, 0–9 points) has been developed for the evaluation. Studies were defined as high quality if they had more than seven points; as medium quality if they had between four and six points; and as poor quality if they had fewer than four points. Studies with NOS score above 4 points were included in the final analysis.

### Statistical analysis

The quantitative data were entered into Cochrane Review Manager (RevMan, software version 5.1, Copenhagen, Denmark: The Nordic Cochrane Center, The Cochrane Collaboration, 2011). The pooled OR with 95% CI was calculated by a fixed-effects model or a random-effects model according to the heterogeneity. P < 0.05 was considered statistically significant on the test for overall effect. The I^2^ statistic was calculated to assess heterogeneity between studies (P < 0.1 was considered representative of significant statistical heterogeneity). If there was heterogeneity between studies, a random-effects model was applied to the data. Alternatively, a fixed-effects model was used for pooling the data. Genotype contrasts, including CC versus TT, CT versus TT, and CC versus CT were analyzed. The most appropriate genetic model was chosen for further analyzed. We also performed subgroup analysis by ethnicity. Harbord’s test^45^ and Peter’s test^46^ were employed to quantitatively assess publication bias (P < 0.05 was considered representative of significant statistical publication bias).

## Additional Information

**How to cite this article**: Chen, G. *et al.* Pharmacogenetics of Complement Factor H Y402H Polymorphism and Treatment of Neovascular AMD with Anti-VEGF Agents: A Meta-Analysis. *Sci. Rep.* **5**, 14517; doi: 10.1038/srep14517 (2015).

## Figures and Tables

**Table 1:** Characteristics of the studies included and qualiyt scores in the current meta-analysis.

Study group (year)	Design	Location	Ethnicity	Treatment	Number of patients	Mean age (years)	Follow-up (months)	Quality score
Brantley (2007)	Retrospective	United States	Caucasian	BVZ	86	79.8	6	6
Dikmetas (2013)	Prospective	Turkey	Undeclared	RBZ	193	71	6	8
Hagstrom (2013)	Prospective	United States	Caucasian	RBZ or BVZ	834	78.5	12	7
Hautamäki (2013)	Prospective	Finland	Caucasian	BVZ	96	78	3.5	7
Kitchens (2013)	Retrospective	United States	Caucasian	RBZ or BVZ	97	80	4	8
Kloeckener-Gruissem (2011)	Retrospective	Switzerland	Caucasian	RBZ	122	78.9	12	7
McKibbin (2012)	Prospective	United Kingdom	Caucasian	RBZ	104	81.5	6	7
Menghini (2012)	Retrospective	Switzerland	Caucasian	RBZ	98	79.3	24	7
Nischler (2011)	Prospective	Austria	Caucasian	RBZ	197	76.9	11.3	8
Orlin (2012)	Retrospective	United States, South Korea	Undeclared	RBZ and/or BVZ	143	80.6	24	7
Park (2013)	Prospective	South Korea	East Asia	RBZ	269	69.5	5	8
van Asten (2014)	Prospective	Netherlands, Germany, Canada	Caucasian	RBZ	391	N/A	3	7
Yamashiro (2012)	Retrospective	Japan	East Asia	RBZ	74	75	12	6

Abbreviations: RBZ, ranibizumab; BVZ, bevacizumab; N/A, not available.

**Table 2:** Genotype distributions of the CFH Y402H polymorphism in studies included in the current meta-analysis.

Study group (year)	Definition of a good response	CFH Y402H genotype					
		Number of patients with good response (%)			Total number of patients		
		CC	CT	TT	CC	CT	TT
Brantley (2007)	Improved visual acuity	2 (16.7%)	31 (54.4%)	5 (50.0%)	19	57	10
Dikmetas (2013)	Gain of 5 or more letters	15 (23.4%)	51 (79.7%)	30 (93.8%)	64	97	32
Hagstrom (2013)	Gain of 3 or more lines	76 (28.1%)	116 (29.7%)	59 (34.1%)	270	391	173
Hautamäki (2013)	Retinal exudate resolved (measured by OCT)	17 (50.0%)	20 (40.0%)	7 (58.3%)	34	50	12
Kitchens (2013)	Retinal exudate resolved (measured by OCT)	3 (20.0%)	11 (21.6%)	6 (19.4%)	15	51	31
Kloeckener-Gruissem (2011)	Gain of 11 or more letters	9 (27.3%)	34 (59.6%)	16 (50.0%)	33	57	32
McKibbin (2012)	Gain of 5 or more letters	14 (56.0%)	30 (56.6%)	9 (34.6%)	25	53	26
Menghini (2012)	Gain of 5 or more letters	10 (37.0%)	28 (66.7%)	17 (58.6%)	27	42	29
Nischler (2011)	Gain of 3 or more lines	6 (12.5%)	22 (23.4%)	13 (23.6%)	48	94	55
Orlin (2012)	Visual acuity improved or unchanged	27 (64.3%)	42 (62.7%)	19 (55.9%)	42	67	34
Park (2013)	Gain of 8 or more letters	0 (0.0%)	24 (47.1%)	108 (50.0%)	2	51	216
van Asten (2014)	Loss of visual acuity < 30% of letters	112 (86.2%)	165 (88.2%)	67 (90.5%)	130	187	74
Yamashiro (2012)	Retinal exudate resolved (measured by OCT)	3 (100%)	10 (52.6%)	34 (65.4%)	3	19	52
All studies		294 (41.1%)	584 (48.0%)	390 (50.3%)	712	1216	776

**Table 3:** Results of meta-analysis for the CFH Y402H polymorphism and treatment response of neovascular age-related macular degeneration.

Polymorphism	Studies by ethnicity (n)	Odds ratio	95% confidence interval	P for test	Model	P for heterogeneity
CC versus TT	Overall (13)	0.55	0.31 to 0.95	0.03	Random-effects model	0.0004
	Caucasian (9)	0.68	0.51 to 0.90	0.008	Fixed-effects model	0.19
	East Asian (2)	0.90	0.18 to 4.55	0.90	Fixed-effects model	0.18
CT versus TT	Overall (13)	0.93	0.68 to 1.28	0.65	Random-effects model	0.06
	Caucasian (9)	0.99	0.77 to 1.28	0.96	Fixed-effects model	0.50
	East Asian (2)	0.80	0.47 to 1.37	0.42	Fixed-effects model	0.51
CC versus CT	Overall (13)	0.60	0.40 to 0.91	0.02	Random-effects model	0.002
	Caucasian (9)	0.61	0.38 to 0.96	0.03	Random-effects model	0.009
	East Asian (2)	1.23	0.24 to 6.16	0.80	Fixed-effects model	0.13
